# Anterior Maxillary Dentigerous Cyst

**DOI:** 10.5005/jp-journals-10005-1041

**Published:** 2009-04-26

**Authors:** Monika Rohilla, Nikhil Marwah, Rishi Tyagi

**Affiliations:** 1 Assistant Professor, Government Dental College, Rohtak, Haryana, India; 2 Reader, Mahatma Gandhi Dental College and Hospital, Jaipur, Rajasthan, India; 3 Assistant Professor, ITS Dental College, Ghaziabad, Uttar Pradesh, India

**Keywords:** Dentigerous cyst, enucleation.

## Abstract

Dentigerous cyst is a development odontogenic cyst, which apparently develops by accumulation of fluid between reduced enamel epithelium and the tooth crown of an unerupted tooth. It is one of the most prevalent types of odontogenic cysts associated with an erupted, developing or impacted tooth, particularly the mandibular 3rd molars, the other teeth that are commonly affected are maxillary canines and third molars. The present case report describes the surgical enucleation of a dentigerous cyst involving permanent lateral incisor, the surgery was followed by oral rehabilitation.

## INTRODUCTION

A cyst is defined as a pathological cavity lined by epithelium. The epithelium itself is surrounded by fibrocollagenous connective tissue and may be derived from various sources. Odontogenic cysts are derived from the odontogenic epithelium which is derived from the basal epithelium of the stomodeum.^1^ Dentigerous cyst is defined as a cyst that originates by separation of the follicle from around the crown of an unerupted tooth. Because the histopathologic appearance of the lining epithelium is not specific, the diagnosis relies on the radiographic and surgical observation of the attachment of the cyst to the cementoenamel junction^2^ The dentigerous cyst is reported to be one of the most common lesions of the jaws. Clinically, dentigerous cysts are usually asymptomatic, but have the potential to become extremely large and cause cortical expansion and erosion.^3^,^4^

## CASE REPORT

**Fig. 1.**
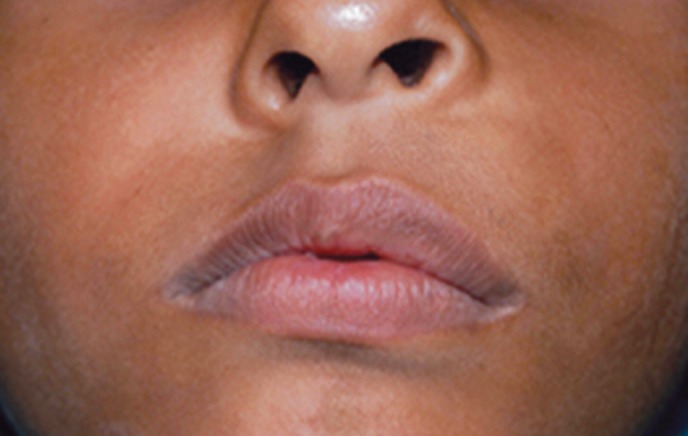
Extraoral view of the patient

A nine year old male patient presented with a progressively enlarging painles swelling on the left side of the face over past 3 months (Fig. 1) which caused elevation of the nasal floor and was also palpable on the palatal surface.

**Fig. 2.**
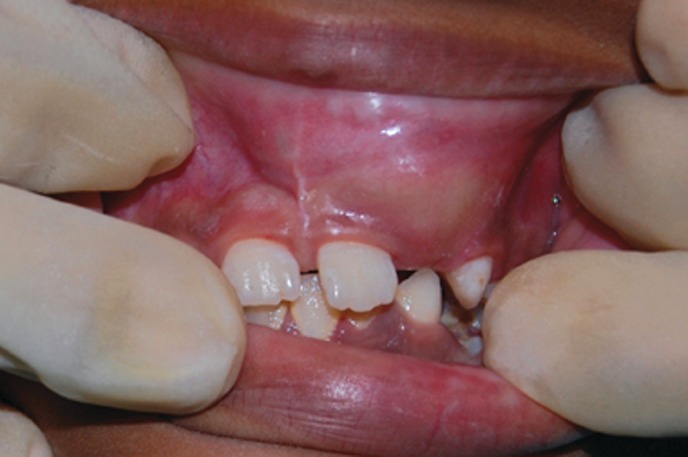
Intraoral view showing bony swelling

**Fig. 3.**
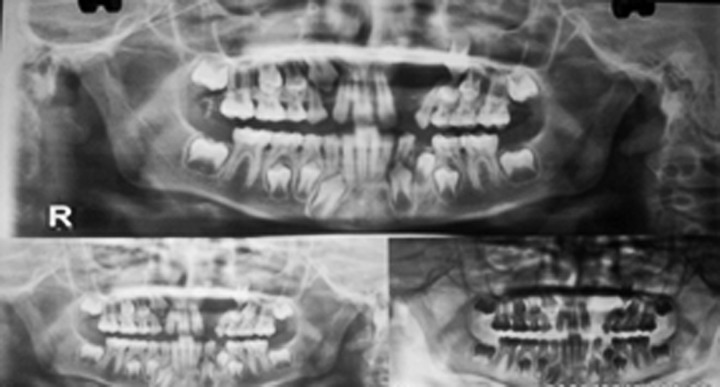
Orthopantomograph showing location of the cyst

**Fig. 4.**
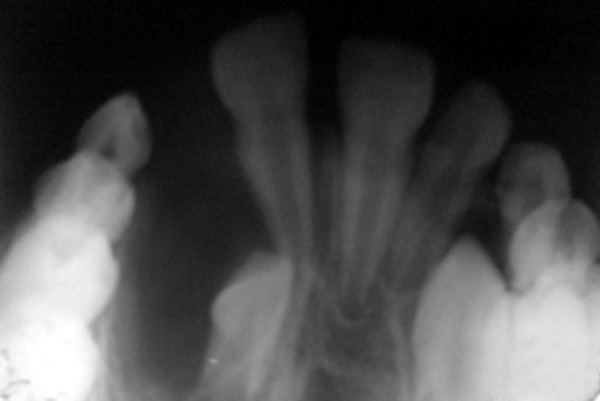
Occlusal view showing extent of bone involvement

**Fig. 5.**
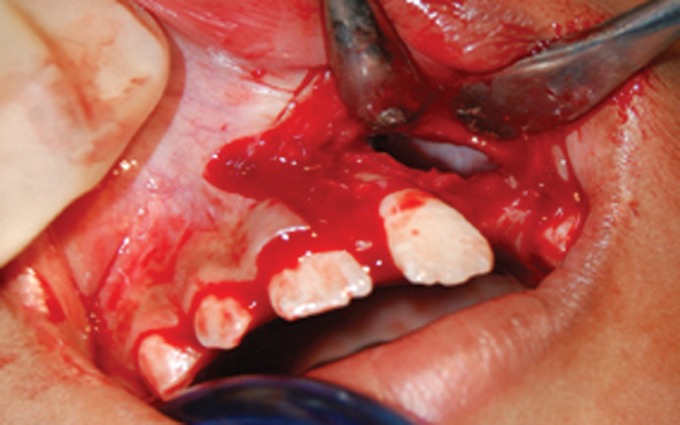
Flap raised with cyst lining ruptured

**Fig. 6.**
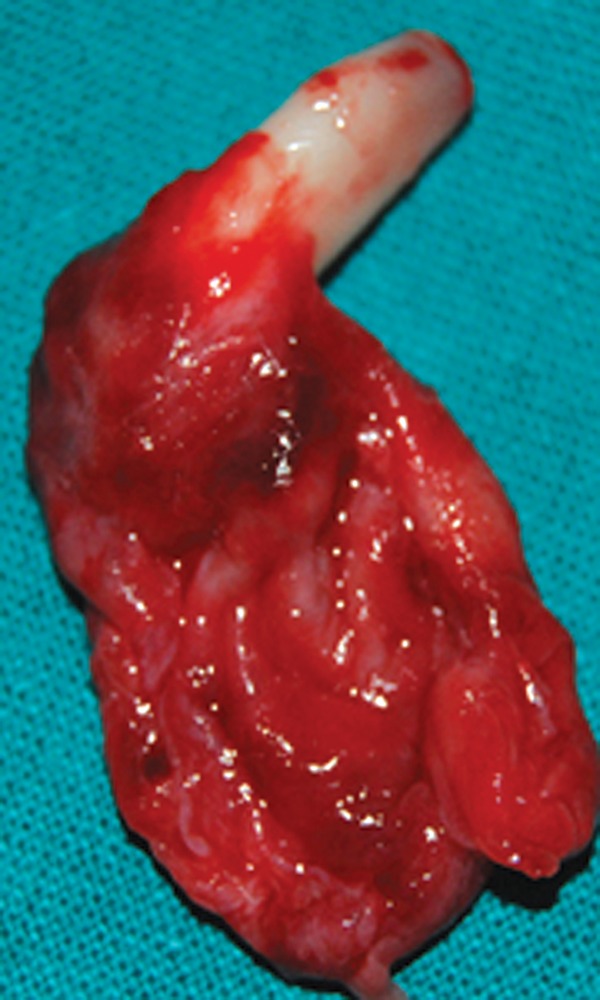
Enucleated cyst with associated tooth

**Fig. 7.**
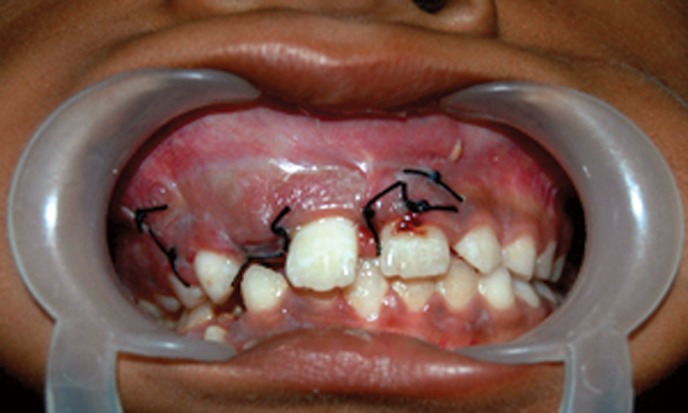
Flap sutured back in position

**Fig. 8.**
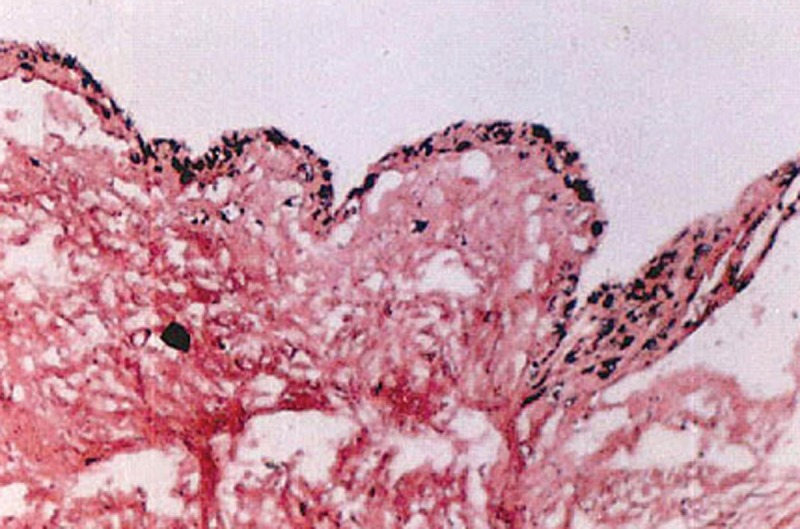
Histological examination of the cyst lining

**Fig. 9.**
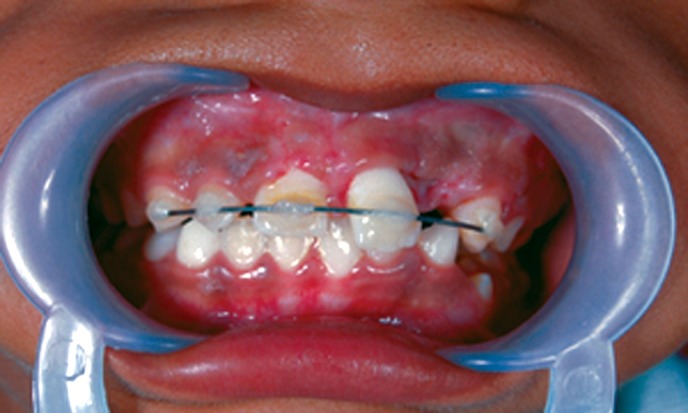
Semi rigid composite wire splint given to immobilize central incisor

**Fig. 10.**
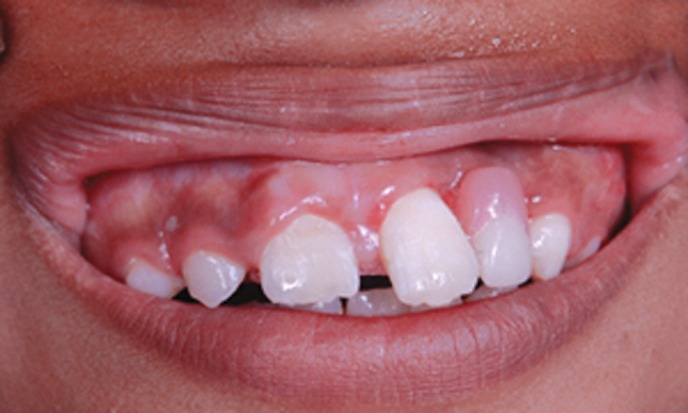
Removable partial denture given to restore aesthetics and occlusion

Clinical examination revealed a firm swelling fixed to the alveolar process of the maxilla (Fig. 2). Diagnostic maxillary occlusal radiograph (Fig. 3) and an orthopantomograph (Fig. 4) showed a radiolucent lesion in the alveolar process of the anterior maxilla. The lesion was well defined measuring approximately 3 × 2.5 cm. Lateral incisor was associated with the lining of the cyst and tooth was displaced. The contents of the swelling were aspirated and sent for investigation, the result of which was consistent with the diagnosis of cystic lesion. After clinical and radiological examination a provisional diagnosis of dentigerous cyst was made. Prior to surgery, routine blood and urine examination were advised. The results were within normal limits. Surgical enucleation of the cyst was chosen as the treatment of choice (Figs 5 to 7). The surgery was done using local anesthesia and under antibiotic cover. The cyst was attached to the cementoenamal junction of left lateral maxillary permanent teeth. The specimen was sent for histopathological examination. The histological examination showed a thin fibrous cystic wall lined by a 2 to 3 layer thick nonkeratinized stratified squamous epithelium with islands of odontogenic epithelium (Fig. 8). The connective tissue showed a slight inflammatory cell infiltrate, which confirmed the diagnosis of dentigerous cyst. Following enucleation of the cyst, the patient was recalled after 1 week for suture removal. As the lesion was big involving considerable amount of bone, left central incisor became mobile after the surgery, henceforth a composite wire splint was placed (Fig. 9). After 1 month, a removable partial denture was delivered, which served functional space maintainer, improved esthetics and phonetics (Fig. 10).

## DISCUSSION

Dentigerous cysts are the most common of the developmental odontogenic cysts of the jaws and account for the approximately 20-24% of all the epithelium lined jaw cysts. It develops around the crown of an unerupted tooth by expansion of the follicle when fluid collects or a space occurs between the reduced enamel epithelium and the enamel of impacted tooth. These cysts are often asymptomatic unless there is an acute inflammatory exacerbation and therefore these lesions are usually diagnosed during routine radiograph.

Radiographic examination of a dentigerous cyst shows a well defined unilocular radiolucency often with a sclerotic border, surrounding the crown of an unreputed tooth. Histologically, the dentigerous cyst consists of a fibrous wall lined by nonkeratinized stratified squamous epithelium consisting of myxoid tissue, odontogenic remnants and rarely sebaceous cells.^5^ They are reported to be more common in male subjects, occur most common in the 2nd and 3rd decades of life and to be most often associated with impacted mandibular 3rd molar and maxillary cuspids.^6^ Radiologically well-defined radiolucent lesions with sharp margins occurring in the maxilla and mandible may be odontogenic or nonodontogenic in origin : such as radicular cyst, dentigerous cysts, odontogenic keratocyst, nonodontogenic cysts like simple bone cysts, aneurysmal bone cyst, stafine cyst or even tumors such as ameloblastoma.

Radicular cyst is the most common odontogenic cyst of the maxilla and mandible. Radiologically, it arises from the apex of the root of a carious tooth and is bounded by a thin rim of cortical bone. The differentiating feature of this entity is its relation to the root of a carious tooth.

Odontogenic keratocyst results from cystic degeneration of the enamel organ before the tooth is formed so that the cyst replaces the tooth. It is commonly noted in the mandible. The classical feature of this cyst is the absence of the related tooth.

Nonodontogenic cysts are observed in the region of incisive canal or nasolabial regions. The incisive canal cyst is in the midline located between the roots of the central incisors of maxilla and is characteristically heart shaped. The nasolabial cyst occurs in the soft tissues of the lateral aspect of the nose and upper lip. These cysts are therefore diagnosed by their classical anatomical location. Aneurysmal bone cyst is seen as expansible multilocular radiolucent lesion. CT/MRI may reveal presence of blood or fluid contents in the cyst.^7^

## CONCLUSION

A dentigerous cyst associated with an anterior tooth will result in failure or eruption of the tooth and therefore lead to esthetic and orthodontic problems. Absence of a lateral incisor can have an impact on the psychology of child.

Further esthetic management has to be considered to prevent and psychological trauma to the child. In the present case, esthetic management was done by providing the patient with a removable partial denture, which also serves as a functional space maintainer.
